# An accelerometer-based navigation system provides acetabular cup orientation accuracy comparable to that of computed tomography-based navigation during total hip arthroplasty in the supine position

**DOI:** 10.1186/s13018-020-01673-y

**Published:** 2020-04-15

**Authors:** Tomonori Tetsunaga, Kazuki Yamada, Tomoko Tetsunaga, Tomoaki Sanki, Yoshi Kawamura, Toshifumi Ozaki

**Affiliations:** 1 Department of Orthopaedic Surgery, Okayama University Hospital, 2-5-1 Shikata-cho, Kitaku, Okayama, 700-8558 Japan; 2 Department of Medical Materials for Musculoskeletal Reconstruction, Okayama University Graduate School of Medicine, Dentistry and Pharmaceutical Sciences, 2-5-1 Shikata-cho, Kitaku, Okayama, 700-8558 Japan

**Keywords:** Hip, Navigation system, Total hip replacement, Retrospective study

## Abstract

**Background:**

Inadequate acetabular component orientation is associated with postoperative impingement, dislocation, and accelerated polyethylene wear. Computed tomography (CT)-based navigation systems provide accuracy for total hip arthroplasty (THA) but are not available in all facilities. Accelerometer-based navigation systems are inexpensive, but their accuracy remains undetermined. This study compares the accuracy of cup orientation in THA using CT-based and accelerometer-based navigation systems.

**Methods:**

This retrospective study included 35 consecutive patients (11 males, 24 females; mean age, 65 years) who underwent primary cementless THA via an anterolateral approach in the supine position. Both CT-based and accelerometer-based navigation systems were used simultaneously. The accuracy of cup orientation was compared between the two systems using postoperative CT.

**Results:**

The accuracy of cup inclination was 2.7° ± 2.0° in the CT-based group and 3.3° ± 2.4° in the accelerometer-based group. The accuracy of cup anteversion was 2.8° ± 2.6° in the CT-based group and 3.4° ± 2.2° in the accelerometer-based group. No significant difference was observed in cup inclination (*p* = 0.29) or cup anteversion (*p* = 0.34) between CT-based and accelerometer-based navigation.

**Conclusions:**

The accuracy of cup positioning did not differ significantly between CT-based and accelerometer-based navigation systems.

## Background

Correct implant positioning is important for good long-term results in total hip arthroplasty (THA). Inadequate orientation of the acetabular component is associated with postoperative impingement [1], dislocation [2], accelerated polyethylene wear [3], liner damage, and restricted range of motion [4]. Surgeons use intraoperative navigation systems to improve the accuracy of implant orientation [5–7]. Computed tomography (CT)-based navigation systems that match preoperative images with the actual pelvis use different techniques. These include CT-based 2-dimensional (2D)-3D matching navigation using intraoperative fluoroscopy, CT-based paired-point matching navigation based on the identification and matching of anatomical landmarks, and surface matching using random identification and registration of reference points [8]. These navigation systems are capable of displaying the angle and position in real time, further improving placement accuracy over CT-free navigation and reducing the rates of dislocation, impingement, and revision THA compared to those of conventional methods [9]. However, CT-based navigation systems also have some disadvantages. 2D–3D matching requires radiation exposure because multidirectional fluoroscopic images must be matched with 3D pelvic images reconstructed from preoperative CT data. Paired-point matching navigation is not very accurate in patients with severe deformities [5]. In addition, not all institutions have expensive CT-based navigation systems.

When THA is performed in the lateral decubitus position, the pelvic tilt error is large [10], and inadequate orientation of the acetabular component is a concern; however, when performed in the supine position, this error is smaller [11]. Nevertheless, the use of intraoperative fluoroscopy or X-ray to confirm alignment of the acetabular component is common. Accelerometer-based navigation systems are less expensive than CT-based navigation systems; they do not require preoperative CT, and they display the angle in real time using multiple reference points during surgery. However, the accuracy of cup orientation using accelerometer-based navigation systems remains unclear.

The purpose of this study was to compare the accuracy of cup orientation in THA performed in the supine position using CT-based and accelerometer-based navigation systems.

## Methods

### Patients’ background characteristics

This study was approved by the Ethics Committee of the institution. This was a retrospective review of 35 hips in 35 consecutive patients (24 hips in 24 females, and 11 hips in 11 males) who underwent primary cementless THA via a modified Watson-Jones approach in the supine position (Table 1). The preoperative diagnoses were osteoarthritis for 26 hips, avascular necrosis of the femoral head for 7 hips, and rheumatoid arthritis for 2 hips. According to the Crowe classification [12], the severity of hip dysplasia in hips with osteoarthritis was defined as Crowe grade I in 33 hips and grade II in 2 hips.

**Table 1:** Demographic data

	*n* = 35
Age (years)^a^	65 ± 11.2 (45–85)
Sex: male/female^b^	11/24
Treated side: right/left^b^	24/11
Diagnosis^b^	
Osteoarthritis	26
Osteonecrosis	7
Rheumatoid arthritis	2
Height (m)^a^	1.56 ± 0.1 (1.39–1.77)
Weight (kg)^a^	58.3 ± 12 (36.6–93.4)
BMI (kg/m^2^)^a^	23.9 ± 3.4 (15.7–31.6)
Crowe G1/2/3/4^b^	33/2/0/0
Sharp angle (°) ^a^	41.7 ± 6.9 (26–58)
CE angle (°) ^a^	19.9 ± 19.9 (− 49–63)
Surgical approach^b^	
Modified Watson-Jones	35

*BMI* body mass index, *CE angle* center edge angle

^a^Values expressed as means ± SD (range)

^b^Values expressed as number of patients

### Devices and surgical procedure

A CT-based paired-point matching navigation system (Vector Vision Hip CT-based version 3.5.2; BrainLab, Heimstetten, Germany) was used in this study. Preoperative CT (Discovery CT750HD; GE Medical Systems, Milwaukee, WI, USA) was performed from the pelvis to the knee. Imaging settings were as follows: tube voltage, 120 kV; tube current, 150 mA; slice thickness, 2 mm; and slice pitch, 2 mm. CT images were saved in Digital Imaging and Communications in Medicine (DICOM) format and imported into the navigation system for preoperative planning and intraoperative registration. An accelerometer-based navigation system (HipAlign Supine; OrthAlign, Aliso Viejo, CA, USA) was used in this study.

**Fig. 1:**
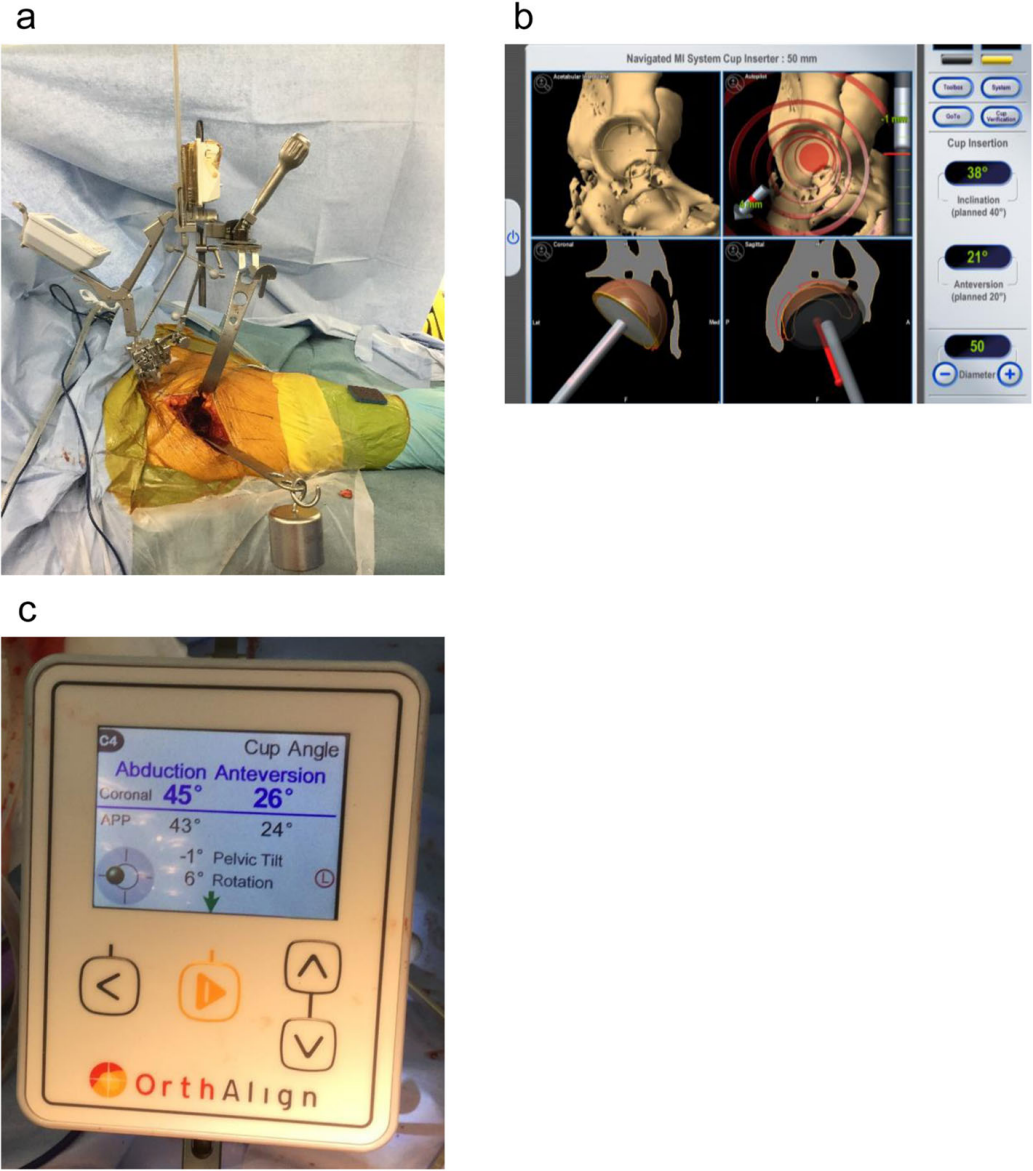
Total hip arthroplasty was performed in each patient with simultaneous CT- and accelerometer-based navigation (**a**). Operative inclination and operative anteversion were displayed on the CT navigation system (**b**), and radiographic inclination and radiographic anteversion were displayed on the accelerometer-based system (**c**). Pelvic tilt and pelvic rotation were also displayed simultaneously on the accelerometer-based system

Surgery was performed by a single operator who had performed ≥ 600 THA procedures. Both accelerometer-based navigation and CT-based navigation were used simultaneously (Fig. 1). During surgery, Schantz screws were inserted into the ala of the ilium on the affected side. A T-shaped reference antenna with three infrared reflection spheres was secured to the Schantz screws. In the registration of the CT-based navigation system, two points on the bilateral anterior superior iliac spines (ASISs), 4 points on the iliac crest, 4 points on the acetabular edge, and 7 points inside the acetabulum were acquired for registration. In the registration of the accelerometer-based navigation, the pelvic base of the accelerometer was fixed with Schantz screws. The sensor was attached, and landmark registration was performed. In table registration, the body axis was aligned with the horizontal axis. Registration of three anterior pelvic plane (APP) landmarks was performed: the bilateral ASISs and the pubic symphysis. After reaming acetabular bone, a cementless hemisphere cup (G7; Zimmer Biomet G.K., Tokyo, Japan) was placed in the reamed acetabulum in an aimed alignment using CT-based navigation. A cementless cup was fixed with three additional screws. The operator measured cup inclination and anteversion of the acetabular component by touching 5 points on its outer margin with a pointer. The operatively defined angles were displayed on the screen of the CT-based navigation (Fig. 1) [13]. The radiographically defined angles were displayed on the screen of the accelerometer-based navigation [13]. Measurements were performed three times, with mean values recorded.

### Evaluations

**Fig. 2:**
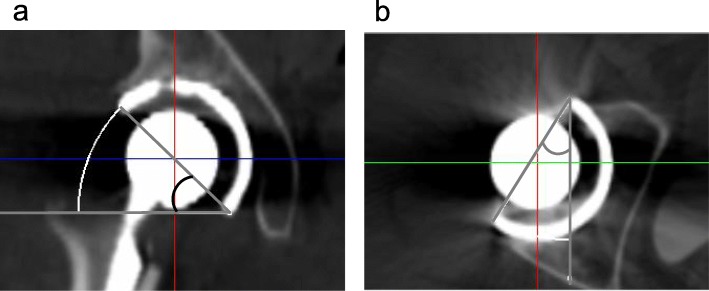
Pelvic coordinate system set to the anterior pelvic plane. Measurement of radiographic inclination on the tomographic coronal plane (**a**) and anatomical anteversion on the tomographic axial plane (**b**)

Postoperative CT data were saved in DICOM format and imported to 3D templating software version 03.08.05 (Kyocera Medical, Kyoto, Japan). First, the pelvic coordinate system was set to the APP in the coronal, sagittal, and horizontal planes. The radiographic cup inclination angle was measured on the slice in which the diameter of the acetabular component in the coronal plane was maximal on the coronal plane (Fig. 2a) [13]. The anatomical anteversion angle was similarly measured in the horizontal plane (Fig. 2b). All measurements were repeated two times each by two orthopaedic surgeons, with mean values calculated. All angles of the acetabular component were given as radiographically defined angles [13]. Accuracy of the acetabular component orientation was defined as the absolute difference between the intraoperative record and postoperative measurements on CT using either CT-based or accelerometer-based navigation [7].

The primary endpoint was to compare the accuracy of cup orientation (absolute difference between the intraoperative record and the postoperative measurement) between the two navigation systems using postoperative CT. The secondary endpoints were intra- and postoperative complications. We also assessed the proportion of patients within the safe zone (i.e., 40° ± 10° inclination; 15° ± 10° anteversion) [14].

### Sample size and statistical analysis

In a pilot study, the mean absolute value of the differences in postoperative measurement from the intraoperative record for cup anteversion was 3.6° with CT-based navigation and 4.3° with accelerometer-based navigation, with a standard deviation of 1.8°. The mean absolute value of the differences in postoperative measurement from the intraoperative record for cup inclination was 2.6° with CT-based navigation and 3.3° with accelerometer-based navigation, with a standard deviation of 1.8°. Based on the effect size in this pilot study, a power calculation (*p* < 0.05; power 0.8) suggested that 33 patients would be needed for a trial. Variables with normal distribution were compared using paired *t* tests, and variables with non-normal distribution were compared using the Wilcoxon signed-rank test. Values are shown as the mean ± standard deviation, and *p* < 0.05 was considered statistically significant. Statistical analysis was conducted using SPSS for Windows version 21 (IBM Corporation, Armonk, NY, USA).

## Results

### Demographic data

Patient background characteristics are presented in Table 1.

### Accuracy of cup orientation and complications

**Fig. 3:**
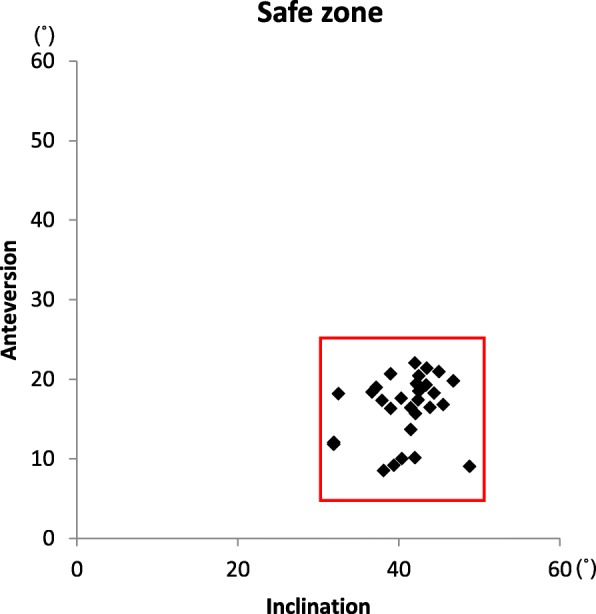
Patients within the safe zone

The navigation systems functioned normally in all cases. Acetabular component orientation during surgery was measured at an inclination of 39.6° (range 32.5°–45.4°) and anteversion of 18.6° (range 11°–32.3°) with CT-based navigation and an inclination of 38.7° (range 29°–50°) and anteversion of 13.9° (range 5°–26°) with accelerometer-based navigation. No significant differences were observed between the CT- and accelerometer-based navigation systems in cup inclination (*p* = 0.16) (Table 2). There was a significant difference in cup anteversion between CT-based and accelerometer-based navigation (*p* < 0.001). The cup inclination on postoperative CT was 40.8° ± 4.1° (31.9°–48.8°), and the anteversion was 16.4° ± 4.0° (8.6°–22.2°). The absolute difference from the values measured on postoperative CT was 2.7° inclination (range 0°–7.2°) and 2.8° anteversion (range 0°–12°) for CT-based navigation and 3.3° inclination (range 0.1°–8.3°) and 3.4° anteversion (range 0.3°–8.5°) for accelerometer-based navigation. The differences from the postoperatively measured values in inclination and anteversion measured with the two systems were not significant (*p* = 0.29, 0.34, respectively, Table 2). The proportion of patients within the safe zone was 100% (35/35 patients, Fig. 3). Loosening of Schantz screws that were used for fixation was not observed. No patients showed postoperative dislocation, fracture, or required repeated surgery for other reasons.

**Table 2:** Intraoperative record and absolute value of differences in postoperative measurement from the intraoperative record for cup angle

		CT-based navigation group (*n* = 35)	Accelerometer-based navigation group (*n* = 35)	*p* value
Intraoperative angles^a^	Inclination (°)	39.6 ± 3.4 (32.5–45.4)	38.7 ± 5.0 (29–50)	0.1565^b^
	Anteversion (°)	18.6 ± 4.7 (11–32.3)	13.9 ± 5.0 (5.0–26)	0.0000^b^
Absolute value of differences^a^	Inclination (°)	2.7 ± 2.0 (0–7.2)	3.3 ± 2.4 (0.1–8.3)	0.2878^b^
	Anteversion (°)	2.8 ± 2.6 (0–12)	3.4 ± 2.2 (0.3–8.5)	0.3366^b^

^a^Values expressed as means ± SD (range)

^b^Paired *t* test

## Discussion

In this study, we performed primary cementless THA in the supine position using CT- and accelerometer-based navigation simultaneously and compared the accuracy of cup orientation between the two systems. The results confirmed that acetabular cup inclination and anteversion were measured with equivalent accuracy using CT- and accelerometer-based navigation.

CT-based navigation is useful for positioning implants in the correct alignment and orientation during THA, and newer navigation systems enable more accurate implant positioning [15]. CT-based navigation can help achieve correct cup orientation irrespective of operator experience [16]. The accuracy of CT-based navigation is such that the absolute error of inclination and anteversion is within approximately 4°, even in patients with severe deformity [5]. In this study, the absolute error of both inclination and anteversion measured with CT-based navigation was < 3°. However, CT-based navigation requires radiation exposure in addition to that from preoperative CT, with increased cost.

Cup malposition, with respect to the Lewinnek safe zone, is an independent risk factor for postoperative dislocation (odds ratio 1.88) [17]. Because the safe zone for cup anteversion is narrower than that for inclination [4], the angle of anteversion must be carefully determined. When THA is performed in the supine position, cup alignment can be monitored with the use of intraoperative fluoroscopy or X-ray. However, surgery should ideally be performed without the use of intraoperative radiation. A factor affecting measurement accuracy using conventional mechanical guidance for THA in the supine position is the variation in pelvic tilt and rotation during surgery compared with the preoperative measurements. Kanazawa et al. [18] reported approximately 3° of pelvic movement in the sagittal, horizontal, and coronal planes during cup positioning. When THA is performed using the HipAlign Supine system, preregistration is performed before the acetabular component is positioned. Because the accelerator within the unit is constantly working, the pelvis is continuously traced. The system displays tilt and rotation of the pelvis with respect to the direction of gravity during surgery, making it more accurate than mechanical guides are, as the accelerometer is unaffected by changes in pelvic tilt during cup positioning [18].

The value of imageless navigation in avoiding radiation exposure has been reported [7, 19]. The mean errors in inclination and cup anteversion with imageless navigation in THA were 6.8° and 3.7°, respectively [15]. Kalteis et al. [19] reported that for patients with anatomical abnormalities such as acetabular dysplasia, imageless navigation imparted a number of disadvantages compared with CT-based navigation. The present study found that in THA performed using the HipAlign Supine system, both inclination and anteversion were highly accurate with no radiation exposure or expensive systems. The HipAlign Supine system has some advantages. Because this system is not optical, the surgeon does not need to worry about his/her positioning. The angle displayed meets the radiographic definition according to both APP and functional pelvic plane (FPP) criteria, even without preoperative CT data.

Although for many years, the APP was considered to be a global reference, it is subject to significant inter-individual variations and variations during positional changes [20]. Reclining the pelvis by 1° leads to a change in functional anteversion of approximately 0.7° [21]. Accordingly, the level of preoperative tilt of the APP must be considered to correctly determine the cup orientation angle [22]. The FPP has been proposed as an alternative to the APP that takes functional pelvic tilt into account during THA [23]. The acetabular component orientation may be adjusted according to pelvic tilt at the time of cup insertion [24–26]. In this study, however, we used the APP rather than the FPP to compare the accuracy between the two navigation systems. This method was used because during surgery, tilt, rotation, and other pelvic parameters should be the same as they are during radiographic assessment. However, the mean intraoperative anterior pelvic tilt was 1.4°, with a mean internal rotation of 1.4° and a mean adduction of 0.9° with the patient in the supine position [11]. A difference in pelvic tilt makes it impossible to perform accurate comparisons of cup alignment on preoperative and postoperative CT images. We therefore used the APP rather than the FPP as the standard plane in this study.

Our study had limitations. Surgery was performed using CT-based navigation as the reference for cup orientation, which was within the safe zone for all patients. However, because we used accelerometer-based and CT-based navigation simultaneously in the same patients, we were able to properly compare accuracy. Second, most patients in this study had Crowe type I or II, with only mild deformity. Severe deformity reportedly reduces the accuracy of CT-based navigation [6]. A study performed in patients with more severe deformity might have obtained a different result. Finally, most patients were comparatively thin, with a mean body mass index of 23.9 kg/m^2^. Factors associated with inadequate cup orientation include the surgical approach; the surgeon's level of experience; a clumsy, low-intervention surgical approach; and obesity [27]. Axial digitization errors affect the anteversion angle by 1.8° for the ipsilateral ASIS, 4.4° for the contralateral ASIS, and 6.8° for the centre of the two pubic tubercles [28]. Pubis registration error thus has a major effect on cup orientation accuracy. An investigation including obese patients might have obtained different results.

## Conclusions

The absolute difference in cup anteversion measured with accelerometer-based navigation was within acceptable limits. The HipAlign Supine provides potential benefit in reducing cup alignment error during THA in the supine position, and its simplicity may contribute to the wider use of computer-assisted orthopaedic surgery.

## Data Availability

All data used and analysed during this study are available from the corresponding author upon reasonable request.

## Abbreviations

THA: Total hip arthroplasty
CT: Computed tomography
2D: 2-dimensional
DICOM: Digital imaging and communications in medicine
ASIS: Anterior superior iliac spine
APP: Anterior pelvic plane
FPP: Functional pelvic plane
